# Novel clinical risk stratification and treatment strategies in relapsed/refractory peripheral T-cell lymphoma

**DOI:** 10.1186/s13045-024-01560-7

**Published:** 2024-06-01

**Authors:** Esther Wei Yin Chang, Ya Hwee Tan, Jason Yongsheng Chan

**Affiliations:** 1 Division of Medical Oncology, National Cancer Centre Singapore, Singapore, Singapore; 2 Duke-NUS Medical School, Singapore, Singapore; 3 Cancer Discovery Hub, National Cancer Centre Singapore, Singapore, Singapore

**Keywords:** Prognosis, Precision Oncology, Epigenetics, Immunotherapy, AITL

## Abstract

Peripheral T cell lymphoma (PTCL) represents a group of heterogeneous hematological malignancies, which are notoriously challenging to treat and outcomes are typically poor. Over the past two decades, clinical prognostic indices for patient risk stratification have evolved, while several targeted agents are now available to complement combination chemotherapy in the frontline setting or as a salvage strategy. With further understanding of the molecular pathobiology of PTCL, several innovative approaches incorporating immunomodulatory agents, epigenetic therapies, oncogenic kinase inhibitors and immunotherapeutics have come to the forefront. In this review, we provide a comprehensive overview of the progress in developing clinical prognostic indices for PTCL and describe the broad therapeutic landscape, emphasizing novel targetable pathways that have entered early phase clinical studies.

## Introduction

Peripheral T cell lymphomas (PTCL) are a rare yet heterogeneous group of non-Hodgkin lymphoma (NHL) that are generally aggressive and confer poor prognosis [1]. They account for 5–10% of all NHL in Western cohorts but are more common in Asia, with incidence ranging from 12 to 22% [2–6]. PTCL comprises of several subtypes that can be identified by shared clinical, pathological and genetic characteristics. The recent WHO classification of Haematolymphoid Tumours Fifth Edition (2022) described nine sub-families/entities of Mature T-cell and NK-cell neoplasms and after excluding entities under the *Mature T-cell and NK-cell leukemias* and *Primary cutaneous T-cell lymphoid proliferations and lymphomas* sub-families, there are still 19 different subtypes of mature T and NK cell lymphomas [7]. The more common, nodal-based subtypes include PTCL not otherwise specified (PTCL-NOS), angioimmunoblastic T-cell lymphoma (AITL) - now renamed as nodal T-follicular helper cell lymphoma, angioimmunoblastic type and anaplastic large cell lymphoma (ALCL) [1]. Prevalence of each subtype varies geographically with PTCL-NOS being more common in Western countries accounting for 20–30% of all PTCLs, while AITL is more common in Asia [8].

Regardless of the subtype, aggressive PTCLs are treated with induction combination chemotherapy followed by consideration for high dose chemotherapy and consolidation autologous haematopoietic stem cell transplant (HSCT) [9–11]. Despite retrospective studies showing a lack of overall survival benefit of anthracyclines in PTCL patients, as with the treatment for their B cell NHL counterparts, CHOP (cyclophosphamide, doxorubicin, vincristine and prednisolone) backbone remains one of the standards for induction chemotherapy of PTCL [11–13]. With the German High-Grade Non-Hodgkin Lymphoma Study Group, Schmitz et al. found that addition of etoposide to CHOP in PTCL patients increased the 3-year event free survival (EFS) of patients age 60 or younger [14]. In patients who are CD30 positive, addition of brentuximab to CHP gave both progression free survival (PFS) and overall survival (OS) benefit in the ECHELON-2 study [15]. Other similar studies attempting to intensify induction treatment by addition of other cytotoxics or novel agents have yet to gain traction.

The role of consolidation autologous HSCT (ASCT) continues to be debated. While it is considered in most transplant-eligible patients with the exception for those diagnosed with ALK-positive ALCL, there is a lack of randomized trial data. Comprehensive Oncology Measures for Peripheral T-Cell Lymphoma Treatment (COMPLETE) study, a large prospective cohort study from the United States, reported that ASCT in nodal PTCL patients in first complete remission (CR1) improved OS and PFS in patients with AITL but not in other PTCL subtypes [16]. ASCT was also found to be associated with better outcomes for patients with advanced stage or intermediate to high International Prognostic Index (IPI) scores. A recent nationwide population-based study of 1427 patients in the Netherlands Cancer Registry also demonstrated superior OS outcomes with the use of ASCT in younger patients < 65 years with advanced stage ALK-negative ALCL, AITL or PTCL [17]. Nonetheless, confirmation of the true benefit of ASCT will probably have to await results of randomized controlled trials [18].

Even despite intensive induction therapies, relapse rates are high and prognosis of PTCL remains poor as compared to their malignant B-cell counterparts. Historically, 5-year PFS for PTCL (excluding non ALK-positive ALCL) is approximately 20% and up to 30% of patients may be primary refractory [19, 20]. The median OS for relapsed/refractory PTCL (R/R PTCL) patients ranged from as low as 2.5 months in the Modena Cancer Registry of 53 patients to 12.3 months in the COMPLETE database for patients with refractory disease and 29.1 months for patients with relapsed disease in the same database [21, 22]. In a 2018 analysis of PTCL patients from the prospective International T-cell Project, out of 937 PTCL patients registered between 2006 and 2016 and who received first line treatment, 68% had relapsed or refractory disease (21% relapsed; 47% refractory). The median survival after relapse in this cohort was 5.8 months. Interestingly, half of the patients with refractory disease did not have high clinical risk scores on diagnosis [23]. With such grave prognosis for R/R PTCL, and the observation that clinical risk scores were not able to identify half the patients who had refractory disease, the need for better prognostic indices was underscored. In this Review, we will consolidate recent advances on the clinical risk stratification and treatment for PTCL and highlight new therapeutic approaches on the horizon.

## Clinical indices for risk stratification of PTCL

### Development of PTCL prognostic indices

They are a myriad of prognostic indices developed for PTCL. The International Prognostic Index (IPI) was first published in 1993 using data from patients with aggressive NHL treated on clinical trials. It was defined by age > 60, serum lactate dehydrogenase (LDH) above upper limit of normal, ECOG performance status (PS) ≥2, stage III or IV and more than 1 extranodal site involvement [24]. Caveats for using the IPI for PTCL patients are firstly, the patient cohort described were trial eligible patients, and secondly there was insufficient immunophenotype data to determine the proportion of PTCL patients in the IPI study cohort. Still, the utility of IPI was demonstrated for PTCL by Gallamini et al. in 2004 and Weisenburger et al. in 2011 [25, 26].

Separately, in 1998, the Groupe d’Etudes des Lymphomes de l’Adulte (GELA) described the prognostic significance of T-cell phenotype and compared the clinical characteristics between B-cell lymphomas (BCL) and PTCL in their NHL cohort recruited under the LNH87 protocol. In multivariate analysis, despite using IPI as a factor, non-anaplastic PTCL remained an independent parameter [27]. In 2004, a revision of the IPI for PTCL unspecified (PTCL-U), Prognostic Index for PTCL (PIT) was developed by the Intergruppo Italiano Linformi (IIL) Lymphoma Registry. Similar to the IPI, multivariate analyses in this cohort found that age, PS and serum LDH were significant clinical factors. Together with bone marrow involvement as a fourth factor, the PIT was developed [25]. It was unsurprising that stage and extranodal involvement failed to retain its prognostic significance in the PIT as compared to BCL, since higher proportion of PTCL patients presented in advanced stages and also with extranodal involvement.

A modified version of the PIT was later described in 2006 using age, PS, LDH and ki-67 scores. This was established in a clinicopathological study that examined expression of 19 markers on tissue microarray of 93 patients, most of whom where in the PIT cohort. On statistical analyses, m-PIT was superior to the IPI or PIT in predicting patient’s survival [28]. While PIT and mPIT had better prognostic ability than the IPI model, it was limited to PTCL-NOS as the other PTCL distinctive entities were excluded. As such, the International Peripheral T-cell Lymphoma Project (IPTCLP) was established across multiple sites in North America, Europe and Asia to study various clinical and pathological indices of PTCL patients [1]. The International T cell index (2005) also known as the IPTCLP score was developed from this large cohort and multivariate analyses found that age, PS and platelet count were independent factors that allowed classification of PTCL-NOS and AITL patients into 4 different risk groups [20]. A comparison of the four prognostic indices for PTCL in a Spanish population by Gutierrez-Garcia et al. in 2011 found the IPTCLP score to be the most significant predictor of OS [29].

### More recent PTCL-NOS indices

Under the IPTCLP, a 2011 retrospective review of 340 PTCL-NOS patients across 22 sites found that while PIT and IPI retained its significance for both OS and failure-free survival (FFS), a statistical review found that PIT was not actually superior to IPI for PTCL-NOS patients. When controlling for IPI and including only bulky disease ≥ 10 cm, platelet count less than 150 × 10^9^/L and transformed tumour cells more than 70%, only the latter was predictive of OS and FFS [26]. Thereafter, a prospective registry of 311 PTCL-NOS patients collected under the T Cell Project was used to develop the T-cell Score in 2017. On multiple Cox proportional hazards regression analysis, the factors that were predictive of OS were that of stage, PS, serum albumin level and absolute neutrophil count (ANC) and these gave rise to three risk groups [30]. The authors also shared that an external validation cohort of patients in the COMPLETE registry found a fair distribution of risk groups and had a comparable discriminant power.

### Prognostic indices for AITL

AITL is the second most common PTCL subtype globally, accounting for 15–20% of PTCL worldwide. From the IPTCLP, a prognostic index for AITL (PIAI) and subsequently an AITL score were developed for AITL patients [31, 32]. In 2013, Federico et al. described the Prognostic Index for AITL (PIAI) from 243 AITL patients from the TCP. Using a combination of age, performance status, extranodal involvement, B symptoms and platelet count, the PIAI was able to differentiate patients into low (0–1 factors) and high-risk (2–5 factors) subgroups [31]. The TCP dataset was expanded and in the updated 282 patient cohort, the authors reported a novel AITL score comprising of age, ECOG performance status, and 2 biochemical markers composed of serum C-reactive protein (CRP) and serum beta 2-microglobulin levels. The AITL score stratified patients to low, intermediate and high-risk groups with 5-year OS estimates of 65%, 54% and 21% respectively. They also found that progression of disease within 24 months (POD24) was strongly prognostic [32].

Like NK/T cell lymphoma (NKTCL), AITL incidence is higher in Asian populations, suggesting ethnogeographic differences in disease biology and justifying the investigation of separate prognostic indices derived from Asian cohorts [33–35]. Tokunaga et al. studied 207 AITL patients in Japan and found that age > 60 years, elevated white blood cell (WBC) and IgA levels, the presence of anemia and thrombocytopenia, and extranodal involvement at > 1 site were significant prognostic factors for OS [36]. In a more recent and simplified analyses, Chang et al. described a novel AITL-PI from a 174 Asian AITL patient cohort, where age > 60, bone marrow involvement, total white cell count > 12 × 10^9^/L and raised serum lactate dehydrogenase were associated with poorer PFS and OS in multivariate analyses. This allowed for a prognostic index (AITL-PI) differentiating patients into low (0–1 factors), moderate (2 factors) and high-risk (3–4 factors) subgroups with 5-year OS of 84.0%, 44.0% and 28.0% respectively. They also validated POD24 as a robust prognostic indicator [37].

### Current limitations

Tables 1 and 2 summarises the known prognostic indices for PTCL and its subtypes. There is certainly a lack of clinical indices that have been cross validated in both Western and Asian populations. The more recently developed indices have allowed for better distribution of patients into the risk groups for a more accurate stratification of each patient’s risk. However, these clinical indices were based on patient populations who were not treated by brentuximab as per the ECHELON-2 study nor did they take into account new molecular prognostic indicators of PTCL and its subtypes (reviewed in [38]). Consequently, the patients who do relapse post induction treatment or are primary refractory remain a challenging group of patients and novel strategies are needed to address their dismal outcomes.

**Table 1:** Clinical indices for risk stratification of PTCL

Clinical Prognostic Indices	International Prognostic Index (IPI)	Prognostic Index for PTCL (PIT)	Modified PIT (m-PIT)	International Peripheral T-Cell Lymphoma Project (IPTCLP) score	T-Cell Score	Prognostic Index for AITL (PIAI)	AITL Score	AITL Prognostic Index (AITL-PI)
**PTCL subtypes**	Aggressive NHL	PTCL-NOS	PTCL-NOS	PTCL	PTCL- NOS	AITL	AITL	AITL
**Clinical factors**	Age > 60 years	Age > 60 years	Age > 60 years	Age > 60 years	Albumin < 3.5 g/dL	Age > 60 years	Age ≥ 60 years	Age > 60 years
	ECOG PS ≥ 2	ECOG PS ≥ 2	ECOG PS ≥ 2	ECOG PS ≥ 2	ECOG PS ≥ 2	ECOG PS ≥ 2	ECOG PS ≥ 2	BM involvement
	LDH > ULN	LDH > ULN	LDH > ULN	Platelet count < 150,000/µL	Stage III/IV	Extranodal sites > 1	CRP > ULN	TW > 12 × 109/L
	Stage III/ IV	BM involvement	Ki-67 ≥ 80%		ANC ≤ 6.5 × 109/L	Positive B symptoms^	β2-M > ULN	LDH > ULN
	Extranodal sites > 1					Platelet count < 150,000/µL		

ECOG PS, Eastern Cooperative Oncolog Group Performance Status; ULN, upper limit of normal; CRP, C-reactive protein; LDH, lactate dehydrogenase; EBV, Epstein-Barr virus; BM, Bone marrrow; β2-M, Beta 2-microglobulin

^Positive B symptoms, any one of night sweats, loss of weight, fever

*Regional lymph nodes involvement was defined as involvement of lymph nodes corresponding to N1-N3 of the primary lesion via the TNM staging system

**Table 2:** Clinical indices and risk-stratified outcomes in AITL

Prognostic Index*	IPI		PIT		PIAI		AITL Score			AITL-PI		
Risk group (score)	Low (0–2)	High (3–5)	Low (0–1)	High (2–4)	Low (0–1)	High (2–5)	Low (0–1)	Int. (2)	High 3–4)	Low (0–1)	Int. (2)	High (3–4)
N (%)	81 (44)	102 (56)	70 (38)	113 (62)	68 (37)	115 (63)	16 (17)	22 (23)	58 (60)	64 (37)	59 (34)	49 (29)
5-year PFS	40%	25%	37%	27%	44%	24%	41%	37%	13%	70%	33%	17%
5-year OS	61%	32%	64%	29%	61%	36%	63%	54%	21%	84%	44%	28%

IPI = International Prognostic Index; PIT = Prognostic Index for T cell lymphoma; PIAI = Prognostic Index for AITL; AITL = Angioimmunoblastic T cell lymphoma; Int. = Intermediate; PFS = progression free survival; OS = overall survival

*cohort and survival data for IPI, PIT, PIAI and AITL Score were retrieved from the International T cell Project study reported by Advani et al. [32] whereas cohort and survival data for AITL-PI was retrieved from the Asian multicentre study by Chang et al. [37]

## Current approved therapies for R/R PTCL

**Fig. 1:**
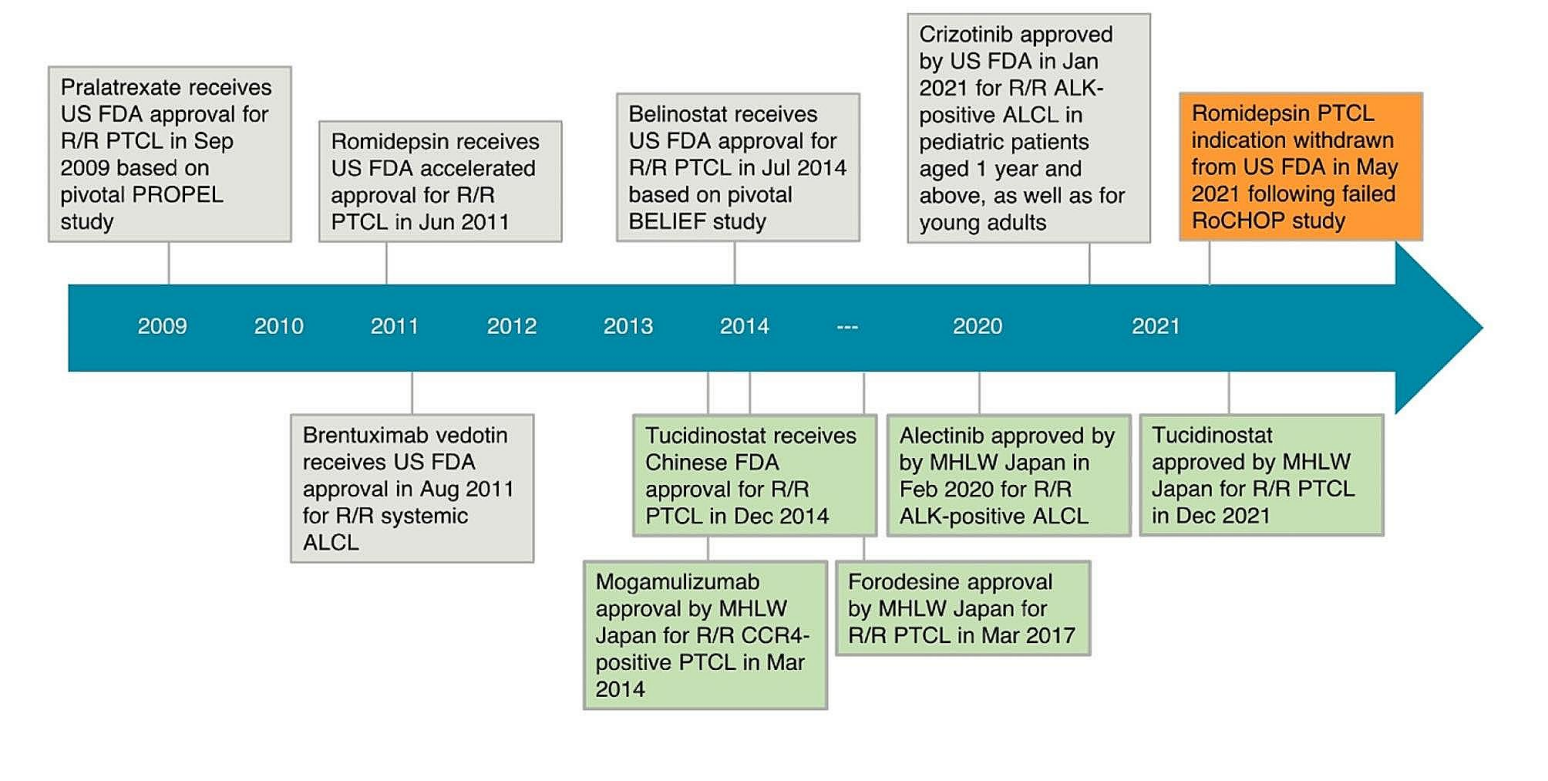
Regulatory approved agents for R/R PTCL

In the setting of R/R PTCL, no established standard of care exists. Frequently, a paradigm involving second-line salvage chemotherapy followed by consolidation with autologous or allogeneic HSCT is adopted as the default approach for the transplant-eligible patients treated outside of clinical trials, with median OS ranging from 12 to 29 months [22, 23]. In transplant-ineligible patients, combinations of chemotherapy and/or novel agents are typically used, albeit with a palliative intent. Several novel agents have demonstrated activity in PTCL and have thus far been approved by national healthcare authorities over the course of two decades (Fig. 1). Notably, retrospective studies suggest a survival benefit in patients who used novel agents over conventional chemotherapy [39, 40].

### Hematopoietic stem cell transplantation (HSCT)

Although salvage chemotherapy followed by consolidation with autologous or allogeneic HSCT remains the only potentially curative approach in R/R PTCL, success is typically achieved in less than 20% of transplant-eligible patients [23, 41]. Furthermore, whether allogeneic transplantation and the accompanied graft-versus-lymphoma effect provide superior outcomes to autologous transplantation remains controversial [42, 43]. Meta-analyses have demonstrated similar survival rates for autologous or allogeneic HSCT, with 5-year PFS of 40–48% and 5-year OS of 53–54% [44]. While outcomes are generally similar, disease biology and patient factors may aid the decision for either approach. In large multicenter retrospective studies evaluating allogeneic HSCT in R/R PTCL, lack of chemosensitivity, older age, and decreased performance status were associated with worse PFS and OS [45–47]. The decision for allogeneic HSCT must be carefully weighed against its higher treatment-related morbidity and mortality rates, and autologous HSCT certainly still represents a reasonable approach, particularly in patients with chemosensitive disease and ALCL histology [48].

### ALK (anaplastic lymphoma kinase) inhibitors – crizotinib and alectinib)

ALK is a receptor tyrosine kinase typically expressed within the central nervous system. Chromosomal translocations, such as t(2;5)(p23;q35), have led to gene fusion events resulting in the oncogenic activation of ALK [49]. In relapsed/refractory ALK-positive ALCL, ALK inhibition has shown efficacy after at least one line of prior cytotoxic therapy. Crizotinib has gained US FDA approval for the treatment of pediatric patients aged 1 year and above, as well as for young adults with R/R ALK-positive ALCL. This approval was based on a single-arm trial of crizotinib in 26 pediatric patients, demonstrating an overall response rate (ORR) of 88% [50]. Similar result were seen in adults in an earlier phase II trial involving 12 patients revealing that crizotinib at a dose of 250 mg twice daily demonstrated an ORR of 84%, with a complete response (CR) rate of 59%. The estimated 2-year PFS and OS rates were approximately 65%. The most common treatment-related adverse events included transient gastrointestinal and mild visual disorders; the most common grade 3 or 4 adverse events was a decrease in neutrophil count [51]. A second-generation ALK inhibitor, alectinib, was evaluated in a Phase II trial in Japan (with subsequent approval), showing a similar ORR of 80%. Adverse events that were more common include oral mucositis, upper respiratory tract infection, maculopapular rash, headache, raised alkaline phosphatase level, constipation and diarrhea [52]. Notably, Alectinib possesses central nervous system penetration and has demonstrated activity [53].

### Brentuximab vedotin

Brentuximab vedotin is an antibody-drug conjugate (ADC) comprised of a chimeric monoclonal antibody (cAC10) linked to an anti-tubulin agent, monomethylauristatin E (MMAE). The monoclonal antibody targets CD30 expressing cells, and MMAE is released intracellularly to bind to tubulin. The activity of BV was initially established in a phase II multicentre trial in relapsed/refractory systemic ALCL. Fifty-eight patients were treated, resulting in an ORR of 86% with a CR rate of 57%. This response was similar in both ALK-positive and ALK-negative ALCL. Furthermore, this favorable response was consistently observed at the 6-year follow-up, with a median duration of response of 26 months. In patients who achieved CR, the 5-year OS was 79%. The most commonly reported adverse effect is peripheral neuropathy [54, 55]. In another Phase II trial examining BV in relapsed/refractory CD30-positive NHL, 35 PTCL-NOS or AITL patients were included. The planned subset analysis revealed an ORR of 41%, with median durations of response and PFS of 8 months and 3 months, respectively. Within this small subset of patients, the AITL subtype demonstrated a better ORR [56].

### Pralatrexate

Pralatrexate is a folate antagonist which selectively enters cells expressing reduced folate carrier type 1 (RFC-1). It inhibits dihydrofolate reductase (DHFR), leading to interruption of RNA synthesis, DNA replication, and elicits apoptosis. Pralatrexate received regulatory approval by the US FDA in 2009, following the PROPEL phase II trial that examined the role of pralatrexate in heavily pretreated R/R PTCL. It resulted in an ORR of 29%, and the median duration of response was 10 months. The most common side effects included cytopenia and mucositis [57].

### Belinostat

Belinostat is a histone deacetylase (HDAC) inhibitor that broadly inhibits all zinc-dependent HDAC enzymes. US FDA regulatory approval was obtained in 2014 based on the BELIEF trial which evaluated belinostat in 129 patients with R/R PTCL, showing an ORR of 26%. Notably, the ORR was higher in the AITL subtype, reaching up to 45%. The median duration of response, PFS and OS were 14 months, 2 months, and 8 months, respectively. The common grade 3–4 adverse events included cytopenia and dyspnea [58].

### Romidepsin

Romidepsin is a bicyclic depsipeptide and a potent inhibitor of class 1 HDAC. Its monotherapy activity was first demonstrated in the pivotal phase II trial in R/R PTCL, encompassing PTCL-NOS, AITL and ALK-negative ALCL. The ORR was approximately 30%, with a median PFS of 20 months. For patients who achieved CR, the median OS was not reached, while it was 18 months for those who achieved PR [59]. This led to US FDA accelerated approval in 2011; however, the approval was subsequently withdrawn in 2021 as the phase III confirmatory trial failed to meet the primary endpoint of improved PFS with Romidepsin plus CHOP compared to CHOP alone in frontline untreated PTCL [60]. Regardless, romidepsin remains as an important and active therapeutic in R/R PTCL that can be considered especially in combination with other novel agents.

### Tucidinostat

Tucidinostat (also referred to as chidamide) is a novel benzamide class of HDAC inhibitors that selectively blocks class I and class IIb HDAC enzymes. In a pivotal phase II study in R/R PTCL, comprising mostly patients with PTCL-NOS, ALCL and AITL, the ORR was 28% and CR rate was 14%. Patients with AITL showed the highest ORR of 50% and a remarkable CR rate of 40% [61]. These findings were confirmed in a real-world studies on R/R PTCL in China where 256 patients who received monotherapy with tucidinostat achieved an ORR of 39% in the overall cohort and 49.2% in the AITL subgroup. Grade 3/4 thrombocytopenia (10.2%) and neutropenia (6.2%) were lower than initially reported in the phase II trial [62]. The safety and efficacy of oral tucidinostat were further examined in a multicenter phase IIb trial in Japan and South Korea, which demonstrated an ORR of 46% across all subtypes. In AITL (*n* = 8), the ORR was reported to be 88%. The median PFS, duration of response and OS were 5.6 months, 11.5 months and 22.8 months, respectively. The most common adverse events included cytopenia and diarrhea [63]. Tucidinostat is currently approved in China and Japan for R/R PTCL.

## Novel treatment approaches

Apart from the limited list of approved therapies for R/R PTCL, several other treatment strategies have been evaluated over the past two decades, though their clinical efficacy has generally been modest. Studies have demonstrated activity of chemotherapeutic agents such as gemcitabine [64–66], platinum agents [67–69], cytarabine-based regimens [70, 71], pentostatin [72–74] and bendamustine [75–77]. Gemcitabine monotherapy has been shown to induce an ORR of 55% in a retrospective cohort of patients (*n* = 20) [66]. Combination with platinum agents such as oxaliplatin [67] or cisplatin [68, 69] is feasible and ORR has been reported to be in the range of 33–72%. In the prospective phase II BENTLY trial (*n* = 60) consisting mainly of patients with R/R AITL (*n* = 32, 53%) and PTCL-NOS (*n* = 23, 38%), the ORR was 50% and CR rate was 28% following three of 6 cycles of bendamustine (at 120 mg/m2 per day on days 1 through 2 every 3 weeks for six cycles). The median PFS and OS were only 3.6 and 6.3 months, respectively [76]. A subsequent retrospective study conducted by the LYSA group demonstrated an ORR of 32.6% and CR rate of 24.6% in a cohort of 138 patients with R/R PTCL, with a dismal median PFS and OS of 3.1 and 4.4 months, respectively [77]. An initial series of five patients with CD30-positive PTCL treated with bendamustine in combination in brentuximab vedotin reported a promising result with 3 CRs observed [78]. A multicenter retrospective study on 82 patients with R/R PTCL from the LYSA centers recently reported ORR of 68% and CR rate of 49% with bendamustine plus brentuximab vedotin. In particular, patients in CR who underwent allogeneic transplant had favorable a PFS of 19.3 months [79]. Brentuximab vedotin has also been combined with the ICE chemotherapy regimen in retrospective studies, eliciting ORR ranging from 29 to 66.7% [80, 81].

Other treatment modalities including immunomodulatory agents, epigenetic agents (Table 3), small molecule inhibitors (Table 4), as well as novel biologics/immunotherapeutics (Table 5) have been explored for the treatment of R/R PTCL and will be summarized in the following sections.

**Table 3:** Novel epigenetic targeted agents and combinations in R/R PTCL

Drug name	Molecular target	Study design, *n*	ORR	CR	Survival outcomes^#^	Reference
Azacytidine	DNMT	Phase III, 86	33%	12%	PFS 5.6 months OS 18.4 months	[98]
Guadecitabine	DNMT	Phase II, 20	40%	10%	PFS 2.9 months OS 10.4 months	[99]
Romidepsin plus ICE	HDAC	Phase I, 18	93%	80%	PFS 10 months OS 15 months	[105]
Romidepsin plus Pralatrexate	HDAC/DHFR	Phase I, 14	71%	29%	PFS 4.4 months OS 12.4 months	[106]
Romidepsin plus Azacytidine	HDAC/DNMT	Phase I, 11	73%	55%	PFS not reached	[110]
Romidepsin plus Azacytidine	HDAC/DNMT	Phase II, 14	54%	38%	PFS 8.0 months OS 20.6 months	[111]
Tucidinostat plus Azacytidine	HDAC/DNMT	Phase I, 19	56%	-	PFS 6.5 months OS 17.5 months	[114]
Tucidinostat plus Parsaclisib	HDAC/ PI3Kδ	Phase Ib/II, 11	67%	56%	-	[115]
Valemetostat	EZH2	Phase I, 57	55%	31%	PFS 7.7 months	[116]
Valemetostat	EZH2	Phase II, 119	52%	27%	PFS 5.5 months OS 17.0 months	[117]
SHR2554	EZH2	Phase I, 28	61%	11%	PFS 11.1 months 12-month OS 92%	[118]
HH2853	EZH2	Phase Ib, 28	61%	21%	3-month PFS 74% 6-month OS 92%	[119]

^#^Median duration, unless otherwise stated

**Table 4:** Novel oncogenic kinase and other small molecule inhibitors in R/R PTCL

Drug name	Molecular target	Study design, *n*	ORR	CR	Survival outcomes^#^	Reference
Dasatinib	SRC kinase	Phase I/II, 9	56%	22%	PFS 2.5 months OS 4.5 months	[125]
Dasatinib	SRC kinase	Phase I, 4	100%	0%	-	[126]
Duvelisib	PI3K-δ/γ	Phase I, 16	50%	19%	PFS 8.3 months OS 8.4 months	[128]
Duvelisib	PI3K-δ/γ	Phase II, 101	49%	34%	PFS 1.5–9.1 months OS 4.8–15.5 months	[129]
Copanlisib	PI3K-α/δ	Phase II, 14	21%	14%	-	[130]
Linperlisib	PI3K-δ	Phase Ib, 43	60%	35%	PFS 10 months 12-month OS 77%	[131]
Linperlisib	PI3K-δ	Phase II, 88	48%	30%	PFS 5.5 months OS 14.2 months	[132]
BR101801	PI3Kγ/δ/DNA-PK	Phase I, 19	32%	21%	PFS 7.5 months	[133]
Duvelisib plus Romidepsin	PI3K/HDAC	Phase Ib, 48	56%	44%	PFS 6.8 months	[134]
Tenalisib plus Romidepsin	PI3K/HDAC	Phase I/II, 16	75%	26%	-	[135]
Copanlisib plus gemcitabine	PI3K	Phase I/II, 25	72%	32%	PFS 6.9 months OS not reached	[136]
Ruxolitinib	JAK1/2	Phase II, 45	27%	7%	PFS 2.8 months OS 26.2 months	[137]
Cerdulatinib (ALXN2075)	SYK/JAK	Phase II, 58	36%	21%	PFS 4.6 months (AITL/TFL)	[138]
Golidocitinib (AZD4205)	JAK1	Phase II, 104	44%	24%	PFS 5.6 months OS 19.4 months	[139]
Alisertib	AURKA	Phase II, 8	50%	-	-	[140]
Alisertib	AURKA	Phase II, 30	30%	7%	-	[141]
Alisertib	AURKA	Phase III, 271	33%	18%	PFS 3.8 months OS 13.7 months	[142]
Bortezomib plus Panobinostat	Proteasome/HDAC	Phase II, 23	43%	22%	-	[143]
Ixazomib	Proteasome	Phase I, 4	25%	0%	-	[145]
Ixazomib	Proteasome	Phase II, 7	14%	14%	-	[146]
Selinexor (ATG-010)	XPO1	Phase I, 2	50%	0%	-	[147]
Selinexor plus ICE	XPO1	Phase I, 10	100%	90%	1-year OS 67%	[148]
Selinexor plus GEMOX	XPO1	Phase Ib, 17	53%	35%	PFS 2.9 months 6-month OS 69%	[149]
Tipifarnib	Farnesyltransferase	Phase II, 42	52%	24%	OS 32.8 months (AITL)	[150]
Forodesine	PNP	Phase I/II, 41	25%	10%	PFS 1.9 months OS 15.6 months	[152]

^#^Median duration, unless otherwise stated; AITL, angioimmunoblastic T-cell lymphoma; TFH, T follicular helper; PNP, purine nucleoside phosphorylase

**Table 5:** Novel immunotherapeutics in R/R PTCL

Drug name	Molecular target	Study design, *n*	ORR	CR	Survival outcomes^#^	Reference
Pembrolizumab	PD-1	Phase II, 13	33%	27%	PFS 3.2 months OS 10.6 months	[153]
Nivolumab	PD-1	Phase I, 5	40%	0%	PFS 14 weeks	[154]
Nivolumab	PD-1	Phase II, 12	33%	17%	PFS 2.7 months OS 6.7 months	[155]
Geptanolimab	PD-1	Phase II, 102	40%	15%	PFS 2.7 months OS 14.6 months	[156]
Mogamulizumab	CCR4	Phase II, 29	34%	17%	PFS 2 months OS 14.2 months	[159]
Denileukin diftitox (diphtheria toxin-IL-2 fusion protein)	CD25	Phase II, 27	48%	22%	PFS 6 months	[162]
E7777 (diphtheria toxin-IL-2 fusion protein)	CD25	Phase II, 17	41%	6%	PFS 2.1 months OS 11.8 months	[163]
Alemtuzumab	CD52	Phase II, 14	36%	21%	-	[169]
AFM13 (bispecific innate cell engager)	CD16A/CD30	Phase II, 108	32%	10%	PFS 3.5 months OS 13.8 months	[172]
Custauzumab (ARGX-110)	CD70	Phase I, 6	-	-	-	[174]
CTX130 (allogeneic CAR-T)	CD70	Phase 1, 7	75%*	-	-	[175]
TTI-621 (SIRPα-IgG1 Fc fusion protein)	CD47	Phase I, 9	22%	-	-	[177]

*at dose level ≥ 3; ^#^Median duration

## Immunomodulatory agents

### Cyclosporine

AITL is a unique subtype of PTCL derived from follicular T-helper (TFH) cells, and is characterized by significant immune dysregulation. Early preclinical studies had suggested the use of calcineurin inhibitor cyclosporine as a potential treatment agent by inhibiting T-cell activation or suppressing differentiation of TFH cells from naïve CD4 + T-cells [82]. In a retrospective series of twelve patients with AITL treated with oral cyclosporine, responses were observed in eight patients (66.7%) with 3 in CR (25%) [83]. A phase II trial in R/R AITL (ECOG 2402) however, was terminated due to slow accrual. Nonetheless in another prospective trial, twelve patients with R/R AITL previously treated with CHOP-like chemotherapy received cyclosporine in combination with prednisolone and high dose intravenous immunoglobulin. In this study, a remarkable ORR of 75% was achieved, including CR rates of 33%. The median duration of response was 20 months and median PFS was 25.5 months [84]. The promising activity of cyclosporine was again suggested in a retrospective literature review of 26 patients with AITL treated with cyclosporine, in which an ORR of 86% was demonstrated beyond the first-line setting. Nonetheless, these data are limited by the small patient numbers and selection bias, thus precluding widespread clinical adoption. Furthermore, although well tolerated, potential toxicity concerns remain including infections, renal insufficiency, and secondary malignancies from immunosuppression.

### Lenalidomide

Lenalidomide is a thalidomide analog and immunomodulatory agent which has been demonstrated to exhibit significant clinical activity in patients with R/R PTCL. In an early phase II study by Dueck et al., 40 patients were treated with lenalidomide at 25 mg daily for 21 days in 4-weekly cycles until disease progression or unacceptable toxicity. In the subcohort of 29 patients with R/R PTCL, the ORR was 24%, while the median PFS was 4 months and OS was 12 months [85, 86]. Another single arm phase II study involved 10 patients with R/R PTCL-NOS who received oral lenalidomide at the same dosing schedule. After the induction phase of four cycles, three patients achieved CR and one patient achieved stable disease, and went on to a maintenance phase using the same regimen. The duration of CR ranged from 11 to 19 months [87]. The EXPECT study was a phase II trial evaluating lenalidomide in 54 patients with R/R PTCL. The ORR was 22%, with a CR rate of 11%, while the median PFS was 2.5 months and median duration of response was 3.6 months. Specifically in the AITL cohort, the ORR was 31% and CR rate was 15%. Median PFS and duration of response were 4.6 months and 3.5 months, respectively [88]. Lendalidomide in combination with brentuximab vedotin has also been investigated in a phase II trial including a small cohort of patients with R/R PTCL, with interim results showing safety and potential efficacy [89].

## Epigenetic therapies

### DNA methyltransferase (DNMT) inhibitors

TFH-derived PTCL, such as AITL, are characterized by recurrent genomic mutations in epigenetic modifier genes such as *TET2*, *DNMT3A* and *IDH2* [90–95], resulting in alterations in their DNA methylation landscape. A retrospective series of 12 patients with R/R AITL evaluated the hypomethylating agent 5-azacytidine 75mg/m^2^ daily given subcutaneously for 7 days in 28-day cycles. Remarkably, the ORR was 75%, with a CR rate of 50%. The median PFS and OS were 15 and 21 months, respectively. Half of the patients in this series however, received rituximab due to detection of EBV in serum or tissue [96]. Objective responses were similarly observed, though at a lower rate (40%) in another retrospective series of 15 patients with R/R AITL who received 5-azacytidine alone, and the median PFS was only 1.6 months. The study also suggested that patients who previously had received fewer lines of prior chemotherapy (≤ 2) had better ORR than those treated with > 2 prior lines (80% vs. 20%), and that those who received a full dose (75 mg/m^2^ for 7 days) of 5-azacytidine may achieve better ORR than those who received suboptimal doses (60% vs. 30%) [97]. In the phase III ORACLE study, 86 patients with R/R AITL or nodal follicular helper T-cell lymphoma were randomized between oral 5-azacytidine (300 mg/day for 14 days in 28-day cycles; Asians received 200 mg/day) and investigator’s choice (gemcitabine, bendamustine or romidepsin). The study unfortunately did not meet the primary endpoint based on a prespecified PFS benefit of 12 months in the experimental arm over 5 months in the standard arm (*p* < 0.025). The median PFS reported was 5.6 and 2.8 months, respectively (*p* = 0.0421); median OS was 18.4 and 10.3 months, respectively. The best ORR and CR at 3 months was 33.3% and 11.9% for 5-azacytidine, and 43.2% and 22.7% in the standard arm [98]. Of note, at least one grade 3/4 adverse event occurred in 76.2% patients on 5-azacytidine vs. 97.7% in the standard arm, and at least one serious adverse event occurred in 26.2% patients on 5-azacytidine vs. 44.2% patients in the standard arm, possibly accounting for the superior survival endpoints in the 5-azacytidine arm despite lower response rates as compared to the standard arm. While the study endpoint was not met, the favourable safety profile of this drug, along with the observed superior OS outcomes, might still suggest clinical utility in the treatment of R/R AITL and other PTCL of TFH origin. Another hypomethylating agent - guadecitabine, was recently evaluated in a phase II study on 20 patients with PTCL (18 R/R and 2 treatment-naïve; inclusive of 11 AITL and 5 PTCL-TFH). Amongst eight patients who responded to treatment (ORR 40%), 2 CR and 5 PR occurred in patients with PTCL of TFH origin [99].

### Histone deacetylase (HDAC) inhibitors

HDAC inhibitors, either as monotherapy or in combination with other agents, may have superior efficacy in PTCL with a TFH phenotype compared to non-TFH subtypes. In a recent study on 127 patients, the ORR was significantly higher at 56.5% (TFH phenotype) as compared to 29.4% (non-TFH) [100], supporting prior observations of higher response rates to HDAC inhibitors belinostat, romidepsin and tucidinostat in AITL as compared to PTCL-NOS and ALCL [58, 59, 61–63]. In order to improve response rates and potentially bridge patients through towards stem cell transplantation, romidepsin has been combined with several chemotherapy regimens, with varied results [101–104]. Particularly, in a prospective phase I trial of 18 patients with R/R PTCL, romidepsin plus ICE induced an ORR and CR rate of 93% and 80%, respectively. Nine patients proceeded to HSCT (5 allogeneic, 4 autologous) after treatment [105]. In keeping with this data, a retrospective report including seven patients treated with romidepsin plus ICE demonstrated an ORR of 71.4% and a CR rate of 57.1%; three patients proceeded to HSCT (1 allogeneic, 2 autologous) [81].

Romidepsin has also been investigated in combination with pralatrexate [106] or bortezomib [107] in early phase I studies involving patients with R/R PTCL. Notably, high response rates of 71% were observed for romidepsin plus pralatrexate in a cohort of R/R PTCL (*n* = 14), including 4 achieving CR [106]. More recent studies have suggested potential synergistic activity of HDAC inhibitors and DNMT inhibitors using preclinical models of PTCL [108, 109], and that the combination of both epigenetic targeting agents romidepsin and azacytidine may be a particularly effective treatment strategy in PTCL. Subsequently, a phase I study of orally-administered azacytidine and romidepsin showed higher response rates and longer PFS in patients with PTCL over those with B-cell lymphomas [110]. In a phase II study, 25 patients who were treatment-naïve (*n* = 11) or who had R/R (*n* = 14) PTCL received azacytidine 300 mg once per day on days 1 to 14, and romidepsin 14 mg/m2 on days 8, 15, and 22 every 35 days. The ORR and CR rates were 61% and 48%, respectively. Notably, patients with T-cell lymphoma of TFH origin exhibited superior ORR and CR rates at 80% and 67%, respectively. In patients with R/R disease, the median PFS and OS were 8.0 and 20.6 months, respectively [111]. These results were confirmed in a retrospective real world study on patients with PTCL treated with azacytidine and romidepsin, the majority of whom had R/R disease with prior therapies. The ORR was 76.9% and CR rate was 53% [112].

In a recent multicentre observational study on 548 patients with R/R PTCL conducted in China, tucidinostat-containing combination therapies exhibited significantly superior ORR (73.2%) as compared with tucidinostat alone (58.6%), albeit with similar CR rates (25.4% vs. 21.1%, respectively) [113]. Results of early phase studies have been reported for the combination of tucidinostat plus azacytidine, as well as for tucidinostat plus the selective PI3Kδ inhibitor parsaclisib [114, 115].

### Enhancer of zeste homolog 2 (EZH2) inhibitors

EZH2 is a methyltransferase that catalyzes the transfer of a methyl group from S-adenosyl-L-methionine to lysine 27 on Histone H3 (H3K27) through its SET domain, and has been implicated in several types of lymphoma. Valemetostat is a first-in-class dual inhibitor of EZH1 and EZH2, which preliminary results from a phase I study (*n* = 57) first reported a promising ORR of 55% (with CR rate of 31%) in R/R PTCL [116]. A global phase II study (Valentine-PTCL01) is currently ongoing to evaluate the efficacy and safety of valemetostat monotherapy in patients with R/R PTCL, reporting a preliminary ORR of 52% and CR rate of 27% in 119 efficacy-evaluable patients [117]. Recently, results from a phase I trial on 28 patients with R/R PTCL (17 AITL, 11 PTCL-NOS) treated with selective EZH2 inhibitor SHR2554 demonstrated an ORR of 61%, median PFS of 11.1 months and 12-month OS of 92% [118]. Another selective EZH1/2 dual inhibitor HH2853 elicited an ORR of 60.7% including a CR rate of 21.4% in a phase Ib study in R/R PTCL [119].

## Oncogenic kinase and other novel small molecule inhibitors

### Src family kinase inhibitors

Somatic nonsynonymous G17V mutations in *RHOA* has been reported in approximately 50–70% of AITL and 18% of PTCL-NOS [94, 95, 120], resulting in its binding and phosphorylation of the VAV1 adaptor protein and activation of downstream T-cell receptor (TCR) signalling cascades. Activating *VAV1* mutations and rearrangements have also been similarly described in *RHOA* wild-type cases [121, 122]. On the same note, recurrent oncogenic rearrangements in Src family kinases, including *FYN*-*TRAF3IP2* and *KHDRBS1-LCK* [123, 124], have also been reported in AITL and PTCL-NOS [120]. Dasatinib, a multikinase inhibitor, is a known inhibitor of Src family kinases and has also been demonstrated to inhibit VAV1 signalling in preclinical models [122, 124]. In a phase I/II clinical trial in patients with R/R non-Hodgkin lymphomas, two CR and 2 PR were observed in PTCL-NOS (*n* = 7) and one patient with AITL had a PR to dasatinib treatment [125]. In a small study involving patients with R/R AITL, dasatinib resulted in PR in all evaluable patients (*n* = 4) after 30 days of treatment. Two patients had a sustained PR beyond 60 days, and one of them went on to receive allogeneic peripheral blood stem cell transplantation [126].

### Phosphatidylinositol 3-kinase (PI3K) inhibitors

The phosphatidylinositol 3-kinase (PI3K) pathway has been shown to be active and potentially targetable in PTCL [127]. Several inhibitors of the phosphatidylinositol 3-kinase (PI3K) have demonstrated clinical activity in patients with R/R PTCL. A phase I study including 16 patients with R/R PTCL showed an ORR of 50% with three patients achieving CR to duvelisib, an oral inhibitor of PI3K–δ/γ isoforms [128]. In the phase II PRIMO study, interim analyses of 101 patients with R/R PTCL included in the study demonstrated an ORR of 49% to duvelisib, which was maintained even in patients with 3 or more prior lines of therapy. The CR rate was 34% and median duration of response was 7.7 months [129]. Copanlisib, a pan-class I PI3K inhibitor with predominant activity against the α/δ-isoforms, demonstrated ORR of 21.4%, including two patients in CR, in a phase II study on R/R non-Hodgkin lymphoma which included 14 evaluable patients with R/R PTCL [130]. Linperlisib, an oral selective inhibitor of the PI3K-δ isoform, was studied in a phase Ib study on 43 patients with R/R PTCL. The reported ORR was 60% and CR rate was 35%, with a promising median duration of response of 15 months and PFS of 10 months [131]. Efficacy was subsequently confirmed in a phase II study, demonstrating an ORR of 48% and CR rate of 30%, with median PFS of 5.5 months and OS of 14.2 months [132]. BR101801, a triple inhibitor of PI3Kγ/δ and DNA-PK, demonstrated in a phase I study an ORR of 32% and CR rate of 21% [133]. Combination therapies incorporating PI3K inhibitors with romidepsin [134, 135] or with gemcitabine chemotherapy [136] have also demonstrated early promising results.

### JAK/STAT inhibitors

In a phase II study of R/R PTCL and mycosis fungoides, the JAK1/2 ruxolitinib showed modest efficacy in PTCL-NOS (ORR 18%, CR 9%), AITL/PTCL-TFH (ORR 33%, CR 11%), and ALCL (ORR 25%, CR 25%) [137]. Cerdulatinib (ALXN2075) is an orally-active reversible ATP-competitive dual SYK/JAK inhibitor with clinical efficacy in R/R PTCL. In 58 evaluable patients in a phase II study, cerdulatinib resulted in an ORR of 36.2% and CR rate of 20.7%, with the majority of responses seen in patients with AITL/PTCL-TFH (*n* = 27; ORR 51.9%, CR 37%). No responses were observed in the 9 patients with PTCL-NOS [138]. Golidocitinib (AZD4205), a rationally-designed oral JAK1-specific inhibitor, was recently evaluated in a multinational phase 2 study (JACKPOT8) on R/R PTCL. Amongst 104 assessable patients, the ORR was 44.3%, including CR rate of 24% [139].

### Aurora kinase inhibitors

The Aurora A kinase inhibitor, alisertib, was evaluated in early phase II studies. Alisertib was administered orally at 50 mg twice daily for 7 days in 21-day cycles. In one study including eight patients with R/R PTCL, the ORR was 50%, with 3 patients (2 in CR and 1 in PR) continuing therapy beyond 1 year [140]. A follow-up phase II study on patients with R/R PTCL or transformed mycosis fungoides, demonstrated an ORR of 30% and 0%, respectively [141]. With these promising results, the phase III Lumiere study investigated alisertib in comparison to investigators’ choice (pralatrexate, gemcitabine, or romidepsin) in R/R PTCL. The ORR for alisertib was 33% and 45% for the control arm, while median PFS was 115 and 104 days, respectively [142]. However, the trial enrolment was terminated early as alisertib did not demonstrate statistical superior efficacy over its comparators.

### Proteasome inhibitors

The proteasome inhibitor bortezomib was combined with pan-HDAC inhibitor panobinostat in a phase II trial of patients with R/R PTCL. ORR was 43% amongst 23 evaluable patients, with a CR rate of 21.7% [113]. In a small pilot study on elderly patients above the age of 65 years, the combination of bortezomib and pralatrexate showed a partial response in 1 of 2 patients in R/R PTCL-NOS and CR in 1 of 2 patients with R/R AITL [144]. In a phase I study using an investigational intravenous proteasome inhibitor ixazomib, a single partial response was observed amongst 4 patients with R/R PTCL [145]. A novel oral formulation of ixazomib was studied in a small phase II trial on patients with R/R PTCL of various histologies (*n* = 7). A single patient with PTCL-NOS achieved a CR, and examination of primary tissue specimens revealed significant loss of intranuclear NF-κB and GATA-3 expression [146].

### Nuclear export inhibitors

Selinexor is an orally-available, potent selective inhibitor of nuclear export through the binding of the nuclear export protein XPO1. A phase I study of selinexor in R/R non-Hodgkin lymphomas showed a partial response in 1 of 2 patients with PTCL [147]. In addition, a phase I study examined the efficacy of selinexor in combination with ICE, in which the ORR and CR rates were 100% and 90%, respectively, amongst 10 evaluable patients with R/R PTCL (*n* = 9) and NKTCL (*n* = 1) [148]. The TOUCH phase Ib study investigated selinexor plus chemotherapy of investigator’s choice (GEMOX or ICE) in patients with R/R PTCL and NKTCL. In patients treated with selinexor plus GEMOX (*n* = 17), ORR was 52.9% and CR rate was 35.3%. ORR of PTCL-NOS (*n* = 8), AITL (*n* = 3), ALCL (*n* = 1) were 62.5%, 0%, and 100%; CR rates were 37.5%, 0%, and 100%, respectively [149].

### Farnesyltransferase inhibitors

Both AITL and PTCL-NOS harboring the CXCL12 rs2839695 A/A genotype (PTCL-CXCL12+) express high levels of CXCL12, a chemokine essential for T-cell chemotaxis to lymphoid organs [150]. Preliminary results of a phase II study showed that tipifarnib, a selective inhibitor of the farnesyltransferase enzyme which aids CXCL12 secretion, elicited a 56.3% ORR and 28.1% CR rate in AITL (*n* = 32). In the 10 patients with PTCL-CXCL12+, the ORR was 40%, including 1 CR. Notably, the median OS for patients with AITL was 32.8 months [151].

### Purine nucleoside phosphorylase inhibitors

Forodesine, a novel purine nucleoside phosphorylase inhibitor, was investigated in a phase I/II study in Japanese patients with relapsed PTCL. In 41 evaluable patients who received oral forodesine at 300 mg twice daily, the ORR was 25% and CR rate was 10%. Median PFS and OS were 1.9 and 15.6 months, respectively [152]. Following this study, forodesine received regulatory approval in Japan for treatment of R/R PTCL.

## Immunotherapeutics

### Immune checkpoint inhibitors

In a single-arm phase II trial on patients with R/R PTCL treated with intravenous pembrolizumab 200 mg every 3-weekly, the ORR was 33% with a CR rate of 27%. CR was observed in 1 case each of ALCL and transformed mycosis fungoides, and 2 cases of PTCL of TFH origin. The median PFS and OS were 3.2 and 10.6 months, respectively. Although overall activity was modest, two of 4 patients in CR remained in remission for more than 15 months [153]. These findings mirror that of an earlier phase I dose-escalation, cohort-expansion study on nivolumab which included 5 cases of R/R PTCL, in which two patients achieved partial responses with duration of 10.6 and over 78.6 weeks respectively [154]. A small phase II study on nivolumab monotherapy in R/R PTCL (*n* = 12) further demonstrated a modest activity, with ORR of 33% and CR rate of 16.5%. The PFS and OS were 2.7 and 6.7 months, respectively [155]. However, due to four cases of possible hyperprogression (defined as time-to-treatment failure of less than or equal to one month from initiation of therapy), the study was stopped prematurely. A larger multicenter phase II study (Gxplore-002) evaluated another anti-PD1 antibody geptanolimab in 102 patients with R/R PTCL, reporting an ORR of 40.4% and CR rate of 14.6%. In this study, treatment efficacy was higher in patients with PD-L1 expression of ≥ 50%, compared to < 50% (ORR 53.3% vs. 25.0%; PFS 6.2 vs. 1.5 months, respectively). Responses were seen across all nodal PTCL subgroup including ALK-negative ALCL (53.8%), ALK-positive ALCL (42.9%), PTCL-NOS (17.9%), and AITL (50%) [156]. No cases of hyperprogression were reported. Future studies will be required to optimize the use of immune checkpoint inhibitors either as monotherapy or in combination with other agents in PTCL.

### CCR4-directed therapy

CC chemokine receptor 4 (CCR4) is a member of the chemokine receptor family and is a surface protein expressed mainly on T-helper and T-regulatory cells, where it aids in cellular migration (chemotaxis) to tissue sites of inflammation [157]. CCR4 was identified to be expressed in several subtypes of nodal PTCL, notably in ALK-negative ALCL (67%), PTCL-NOS (38%) and AITL (35%) [158]. Mogamulizumab, a glyco-engineered anti-CCR4 antibody, targets CCR4 and induces an antibody-dependent cellular cytotoxicity reaction leading to tumor cell death and lysis [157]. A phase II trial for mogamulizumab conducted in Japan identified an ORR of 34% in their cohort of patients with R/R CCR-positive PTCL, inclusive of PTCL-NOS (*n* = 16), AITL (*n* = 12) and ALK-negative ALCL (*n* = 1). Subgroup responses were 19%, 50% and 100%, respectively. Median PFS and OS were 2 and 14.2 months, respectively. CCR4 expression was determined by immunohistochemistry and was classified according to the proportion of stained tumor cells (at least 10%). No significant correlation between CCR4 expression levels and response rates to mogamulizumab was demonstrated [159]. Common adverse events included reversible cytopenias, pyrexia, and skin disorders. Based on these results, mogamulizumab was approved in 2014 for treatment of R/R CCR4-positive PTCL and cutaneous T-cell lymphoma in Japan.

### CD25-directed therapy

CD25, also known as the interleukin-2 receptor α-chain or IL-2Rα, is positively expressed in 40–50% of PTCL [160, 161]. Denileukin diftitox is a recombinant fusion protein linking diphtheria toxin to IL-2. In a cohort of 27 patients with R/R PTCL, denileukin diftitox elicited an ORR of 48.1%, including six patients with CR. In subgroup analysis, the ORR was 61.5% in patients with CD25-positive tumours (≥ 10% tumour cells CD25+) and 45.5% in CD25-negative tumours (< 10% tumour cells CD25+). Median PFS was 6 months [162]. A subsequent phase II study in Japan, investigated E7777, another recombinant fusion protein composed of diphtheria toxin to IL-2 similar to denileukin diftitox, albeit with reported improved purity and increased percentage of active monomer. Amongst 17 patients with R/R PTCL, the ORR was 41.2%, with one patient achieving CR. Responses were observed in both patients regardless of high (≥ 20%) or low (< 20%) levels of CD25 + tumour cells at 45.5% and 33.3%, respectively. The median PFS was 2.1 months and OS was 11.8 months [163].

### CD52-directed therapy

CD52 is a cell surface antigen found on mature lymphocytes, and is variably detectable in various subtypes of PTCL [164–168]. Alemtuzumab, a humanized anti-CD52 monoclonal antibody, was studied in a phase II study of 14 patients with heavily-pretreated PTCL, resulting in an ORR of 36%. Three patients achieved CR. The study was closed as a result of significant toxicities, including cytomegalovirus reactivation, pulmonary aspergillosis, Epstein-Barr virus-related hemaphagocytosis, and pancytopenia. Five treatment-related deaths were reported [169].

### Chimeric antigen receptor (CAR)-T cell therapy and other novel therapeutics

Several promising biologics, such as bispecific antibodies, novel antibody targets, and even CAR-T cell therapy have recently appeared on the horizon for PTCL treatment. This is despite several potential challenges such as fratricide leading to T-cell aplasia, or contamination with malignant T-cells during apheresis for T-cell collection, since normal T-cells share mutual antigens with the malignant T-cells [reviewed in 171]. Just as early preclinical data on CD38/CD3xCD28 trispecific antibody therapy are emerging, along with ongoing CAR-T therapy trials targeting CD5, CD7, CD30, CD70 or TRBC1 that are highly anticipated to advance the immunotherapy landscape in PTCL, we are already witnessing promising results from early trials [171].

AFM13 is a tetravalent, CD16A/CD30 bispecific Innate Cell Engager that binds CD30 on PTCL cells and CD16A on innate effector cells, thereby redirecting and enhancing the innate immune response to the tumor cells. In the REDIRECT phase II study on patients with CD30-positive R/R PTCL, the ORR was 32.4% and CR rate was 10.2%. In histological subgroups, ORR was highest in AITL (53.3%), followed by ALCL (23.1%), and PTCL-NOS (22.0%). Median PFS and OS were 3.5 and 13.8 months, respectively [172]. Other early phase clinical trials have also explored CD70, a transmembrane protein member of the tumor necrosis factor superfamily, as a potential target in PTCL [173]. CD70 expression was demonstrated on various T-cell lymphomas on immunohistochemistry, including nodal PTCL subtypes such as PTCL-NOS and AITL. Potential therapeutic approaches include using a defucosylated anti-CD70 monoclonal antibody (ARGX-110/custauzumab) [174], anti-CD70 allogeneic CAR-T cells (CTX130) [175], and antibody drug conjugates [176]. Another potential novel immunotherapeutic agent is TTI-621 (SIRPα-IgG1 Fc), a novel innate immune checkpoint inhibitor that activates antitumor activity by simultaneously blocking CD47-mediated inhibition of phagocytosis (“don’t eat me” signal) and activating prophagocytic signals through IgG1 engagement of Fcγ receptors on macrophages and natural killer cells. A first-in-human phase I study showed that TTI-621 monotherapy induced a response in two of 9 patients with R/R PTCL, including AITL and PTCL-NOS [177].

## Conclusion

In summary, the progress in the molecular understanding of PTCL has led to a more comprehensive therapeutic landscape that is rapidly evolving towards personalized treatment strategies and targeted therapies depending on specific histology and molecular profile. Current disease subtype-specific risk indices, while useful for prognostication, certainly remain inadequate as tools for patient stratification and treatment selection. We are now perhaps at an inflection point with new opportunities to overcome the unmet needs in the treatment of PTCL, particularly with the rapid emergence of novel biologics such as multispecific antibodies and CAR-T therapies. Ongoing research and clinical trials must continue to explore avenues for improving outcomes in patients with PTCL.

## Data Availability

No datasets were generated or analysed during the current study.

## Abbreviations

PTCL: Peripheral T cell lymphoma
NHL: Non-Hodgkin lymphoma
PTCL-NOS: PTCL not otherwise specified
AITL: Angioimmunoblastic T-cell lymphoma
ALCL: Anaplastic large cell lymphoma
ASCT: Autologous haematopoietic stem cell transplant
CHOP: Cyclophosphamide, doxorubicin, vincristine and prednisolone
PFS: Progression free survival
OS: Overall survival
CR: Complete remission
ORR: Overall response rate
IPI: International Prognostic Index
LDH: Lactate dehydrogenase
PS: Performance status
BCL: B-cell lymphoma
PIT: Prognostic Index for PTCL
FFS: Failure-free survival
PIAI: Prognostic index for AITL
POD24: Progression of disease within 24 months
GELA: Groupe d’Etudes des Lymphomes de l’Adulte
IPTCLP: International Peripheral T-cell Lymphoma Project
IIL: Intergruppo Italiano Linformi
NKTCL: NK/T cell lymphoma
WBC: White blood cell
CRP: C-reactive protein
ANC: Absolute neutrophil count
RFC-1: Reduced folate carrier type 1
DHFR: Dihydrofolate reductase
HDAC: Histone deacetylase
TFH: Follicular T-helper
DNMT: DNA methyltransferase
EZH2: Enhancer of zeste homolog 2
H3K27: Lysine 27 on Histone H3
PI3K: Phosphatidylinositol 3-kinase
